# Concept of a Radiofrequency Device for Osteopenia/Osteoporosis Screening

**DOI:** 10.1038/s41598-020-60173-5

**Published:** 2020-02-26

**Authors:** Sergey N. Makarov, Gregory M. Noetscher, Seth Arum, Robert Rabiner, Ara Nazarian

**Affiliations:** 1 ECE Dept., Worcester Polytechnic Institute, Worcester, MA 01609 USA; 2 Athinoula A. Martinos Center for Biomedical Imaging, Massachusetts General Hospital, Harvard Medical School, Boston, MA 02114 USA; 3 Neva Electromagnetics, LLC., Yarmouth Port, MA 02675 USA; 4 Alnylam Pharmaceuticals, Cambridge, MA 02412 USA; 5 IlluminOss Medical, East Providence, RI 02914 USA; 6 Center for Advanced Orthopaedic Studies, Beth Israel Deaconess Medical Center, Harvard Medical School, Boston, MA 02215 USA

**Keywords:** Musculoskeletal models, Biomedical engineering

## Abstract

Osteoporosis represents a major health problem, resulting in substantial increases in health care costs. There is an unmet need for a cost-effective technique that can measure bone properties without the use of ionizing radiation. The present study reports design, construction, and testing of a safe, and easy to use radiofrequency device to detect osteoporotic bone conditions. The device uses novel on-body antennas contacting the human wrist under an applied, operator-controlled pressure. For the dichotomous diagnostic test, we selected 60 study participants (23–94 years old, 48 female, 12 male) who could be positively differentiated between healthy and osteopenic/osteoporotic states. The band-limited integral of the transmission coefficient averaged for both wrists, multiplied by age, and divided by BMI has been used as an index. For a 100 MHz frequency band centered about 890–920 MHz, the maximum Youden’s J index is 81.5%. Both the sensitivity and specificity simultaneously reach 87% given the calibration device threshold tolerance of ±3%. Our approach correlates well with the available DXA measurements and has the potential for screening patients at risk for fragility fractures, given the ease of implementation and low costs associated with both the technique and the equipment. The inclusion of radiofrequency transmission data does add supplementary useful information to the available clinical risk factors.

## Introduction

Approximately 50% of women and 20% of men over the age of 50 will suffer from a fragility fracture in their remaining lifetime^1^. Hip fracture is one of the most serious and debilitating outcomes of osteoporosis^2,3^ with a 14–36% mortality rate during the first year after the fracture^4^. Hip fracture incidence rates are known to increase exponentially with age in both women and men^5^, and with the rising life expectancy throughout the globe, osteoporosis is expected to increase to 14 million cases with over 47 million cases of low bone mass density by 2020. Thus, the number of fractures is predicted to double or triple by 2040^6^.

The World Health Organization (WHO) has defined individuals at risk for these fractures based on their *areal* bone mineral density (aBMD, g/cm^2^) relative to that of a normal young adult, as measured by Dual-energy X-ray Absorptiometry (DXA). The disadvantages of DXA include: exposing patients to ionizing radiation doses of up to 0.86 mrem^7^; the surrounding soft tissues can introduce relevant measurement errors^8,9^, bone mineral density (BMD) measurements are affected by variations in bone size^10,11^, and cortical and trabecular bone cannot be separated^12^. Additionally, fracture predictions based on aBMD have been shown to be neither sensitive nor specific^13–17^.

Current methods to detect osteoporosis depend on BMD measurements. These exams include DXA, Quantitative Computed Tomography (QCT) and high-resolution peripheral quantitative computed tomography (HR-pQCT, although not in use clinically). DXA, the gold-standard imaging tool, is used to establish or confirm a diagnosis of osteoporosis and to predict future fracture risk. Although it allows for bone mass assessment, it does not provide any information about bone quality, structure or strength^18^. QCT is more sensitive than DXA when assessing BMD by adding information about bone geometry using 3D image reconstructions. Additionally, trabecular and cortical bone compartments can be assessed separately with this technique^19^. However, radiation exposure with this approach is considerably higher when compared to DXA. High resolution pQCT provides superior image resolution, allowing for analysis of bone microstructure and assessment of volumetric bone mineral density (vBMD)^20^. A major disadvantage is increased radiation exposure and unavailability of the system in clinical settings, with approximately 30 institutions in the United States having access to this technology in a research capacity^21^. Therefore, there is an unmet need for a cost-effective technique that can accurately measure bone properties without the use of ionizing radiation.

Quantitative ultrasound has been used as a low-cost, non-ionizing technique to screen patients for osteoporosis, employing a dedicated scanner to acquire data predominantly at the calcaneus. Emitter and receiver probes are brought in close proximity of the heel soft tissue in a water bath or through the application of ultrasound jelly, where the velocity and amplitude of transmission of the ultrasonic waves through bone are measured. It has been observed that speed of sound decreases in osteoporotic bone, while broadband ultrasonic attenuation (calculated in decibels per megahertz) increases. Quantitative ultrasound has been shown to predict osteoporotic fractures^22^; however, the proliferation of the devices with technological diversity, examination of different anatomic sites, and the low correlation of results with DXA^23^ have been problematic in the clinical application of quantitative ultrasound^24^. While quantitative ultrasound differentiates between individuals with and without fragility fractures^25,26^, and can predict fracture risk^27,28^, it has not shown the ability to diagnose osteoporosis in a manner similar to DXA. Therefore, it is currently not recommended to monitor treatment response according to the International Society for Clinical Densitometry (ISCD)^24^, given the lack of large-scale studies outlining monitoring efficacy^29^. Quite recently, another ultrasound approach (Echographic Spectrometry) has been proposed^30^.

A commercial ultrasound device Bindex^®^ uses the pulse-echo technique to measure thickness of the frontal cortical shell of the tibia bone^31–34^ These measurements have been found to correlate well with DXA measurements^31^.

Microwave imaging of (heel) bone was first introduced by Dr. Keith Paulsen and his research group at Dartmouth College approximately ten years ago as an alternative non-ionizing diagnostic method to assess bone health^2,35–38^. Due to the well-known complexity and poor spatial resolution of the standard microwave imaging setup^39,40^, used in these studies, no clinically applicable results have been generated to date. However, the underlying physical idea of this method is simple and powerful. In osteoporosis, bone mass decreases and pore size increases. The lost bone mass is replaced by a mixture of yellow bone marrow. Such substantial changes in physical properties must alter electromagnetic tissue properties^41,42^, and must generate a significantly different radio-frequency (RF) channel through the bone. It may therefore be sufficient to track an integral measure of radio wave propagation along the path through the bone instead of restoring the complete permittivity map of the bone and the surrounding tissues, as attempted previously^2,35–38^.

To do so, we select a body compartment, where bone constitutes a significant fraction of the total tissue volume. The wrist is preferred as it is more easily accessible. We have designed on-body transmitting/receiving dual antiphase patch antennas with controlled pressure on this anatomic site^43^. We have further measured radio wave propagation through this compartment and compared our results with osteoporotic and osteopenic (low bone density) conditions established via DXA and through a history of bone fracture.

Essentially, the present device is equivalent to two low-power cellphones placed on both sides of the wrist with one transmitting and the other receiving. The radiofrequency (RF) signal goes through the bone and mimics its properties. The RF setup radiates into the wrist 0.1 W of RF power in the 0–2 GHz band, which is significantly less than the radiated power of a typical cellphone (between 0.6 W and 3 W) operating in the same frequency band. No gels or water baths typical for ultrasound measurements are required. The proposed system is designed as a means to increase the screening capabilities of the medical community with a simple office based technician driven system.

## Materials and Methods

### Device concept

**Figure 1:**
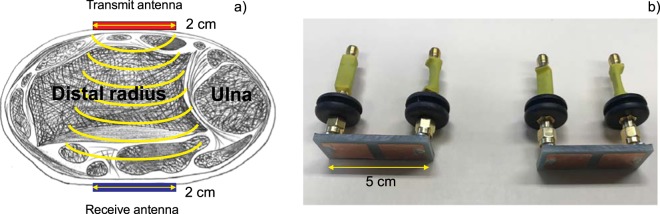
(**a**) Idealized diagram illustrating antenna placement on both sides of a human wrist. (**b**) Transmit and receive dual antiphase patch antennas with individual lumped-component matching networks (green isolation) designed for the present study. Antenna length (along the wrist) is 5 cm; antenna width (across the wrist) is 1.8 cm.

The device concept is illustrated in Fig. 1a. Two dual antiphase patch antennas (Fig. 1b), described in the text below, are placed on both flat sides of the wrist close to the position of the ulnar head under an applied controlled pressure of 1 kg force. The radiofrequency signal in the 0–2 GHz band travels from the transmit antenna through bone, cartilage, and soft tissue to the received antenna while being attenuated and scattered. The total amount of attenuation and scattering is measured via the microwave transmission coefficient ${S}_{21}(f)$ and is correlated to osteopenic and osteoporotic conditions. The antenna width across the wrist is 2 cm; the antenna length along the wrist is 5 cm; facilitating good contact between the two surfaces.

### On-body antenna design

Dedicated antennas for radiating into the body or receiving from the body are located on the skin surface. They are applicable to wireless body area networks, microwave imaging, as well as implanted body sensors. Such antennas include broadband monopoles/dipoles^2,40,44–52^ and their modern printed versions^53–55^ as well as small-size arrays^2,40,44–53,56–59^. More recently, wideband and multiband single patch antennas (slotted or not) have been suggested and investigated^59–65^.

**Figure 2:**
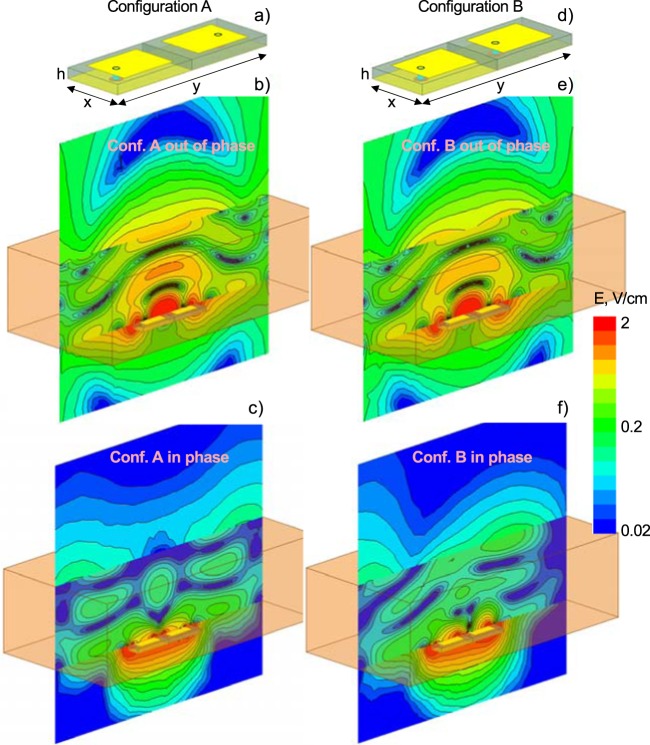
Four representative antenna configurations (configuration A fed in antiphase or phase and configuration B fed in antiphase or phase, respectively). Out-of-phase antenna array concept (**b**,**e**) is compared to the standard in-phase directional antenna array concept (**c**,**f**). Electric field magnitude is shown given 0.05 W of power per individual antenna radiator port at 915 MHz. The dual antiphase patch antenna in (**a**) has been selected.

In the initial device prototypes, single slotted patch antennas^66,67^ or printed dipoles attached to the wrist were employed. Both antenna types suffered from a lower transmission coefficient through the wrist. To overcome this, a new antenna configuration shown in Fig. 2a, as configuration A was designed and optimized^43^. This new configuration resembles an array of two patch antenna radiators in echelon, with the patches facing toward the body. However, the probe (or microstrip) feeds are located on the opposite sides of the patches. Most importantly, the individual antennas are fed in antiphase, i.e. via a 180° power splitter. A similar antiphase feeding mechanism for two dipole wings is otherwise known as the Dyson balun^68–70^.

The two antiphase patch radiators provide a greater penetration depth and transmitted signal into the body than a single antenna or two adjacent patch antennas in phase. To demonstrate this, Fig. 2 presents simulation results for the radiated electric field of four representative antenna configurations. These results are obtained at 915 MHz simulation frequency with the commercial FEM software ANSYS HFSS Electronics Desktop 2019R1. The wrist is modeled as a brick with a height of 6 cm, an average relative dielectric constant ${\varepsilon }_{r}=30$, and an average conductivity $\sigma =0.1\,{\rm{S}}/{\rm{m}}$. The 3.25 mm thick substrate (FR4 or a low-loss Rogers laminate) has the size of $50\times 20\,{\rm{mm}}$. The two radiators are fed in antiphase, with a port power of 0.05 W each. Both ports are matched to $10-j5\,{\rm{ohm}}$. The antenna indicates both parallel and series resonances, which are closely spaced.

While Fig. 2b shows the magnitude of the electric field for antiphase feeding, Fig. 2c is the same result but for the in-phase feed. In the former case, the signal propagates into the body and is strong. In the latter case, the signal is significantly absorbed in the vicinity of the antenna and is mostly directed outwards, i.e. into air. For comparison purposes, Fig. 2e and f show the same results but when the two individual patch antennas are in echelon as in Fig. 2d (configuration B). The antiphase feeding again causes strong transmission, but it is weaker than that of the prime configuration A in Fig. 2b. Furthermore, the beam is not entirely symmetric.

**Figure 3:**
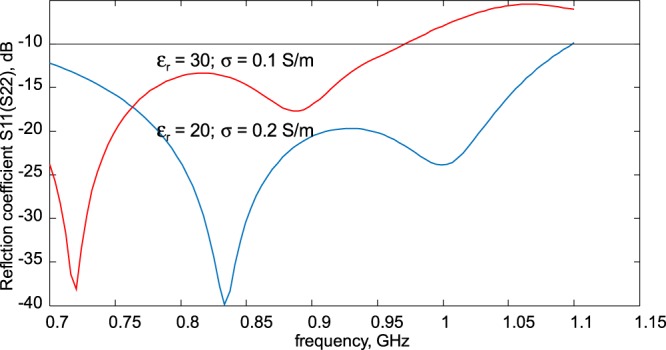
Simulated reflection coefficient magnitude in dB, ${|{S}_{11}(f)|}_{dB}$, of the dual antiphase patch antenna from Fig. 2a as a function of frequency at different values of average tissue permittivity/conductivity. Matching to characteristic impedance of $10-j5\,\Omega$ is assumed.

When matched to $10-j5\,{\rm{ohm}}$ prior to the power splitter, the dual antiphase patch antenna in configuration A from Fig. 2a indicates a sufficiently large impedance bandwidth shown in Fig. 3. The band is centered approximately around 800–900 MHz and holds for different values of both the dielectric constant and the conductivity of the tissue. Therefore, configuration A from Fig. 2a has been selected as the on-body dual antiphase patch antenna prototype.

Numerical simulations indicate that the antenna performance quickly deteriorates when a gap between the antenna and the body reaches or exceeds 1 mm. Therefore, in the ideal setting, this gap should be either minimized or a direct ohmic contact with body surface should be maintained with the assistance of a gel. We suggest minimizing the gap by applying a controlled pressure to the antenna attached to the body as explained below negating the need for a gel.

### Simulation with realistic human phantom

The anatomically accurate computational human model VHP-Female^65^, derived from the Visible Human Project (VHP) of the U.S. National Library of Medicine, has been used for the simulations of a realistic, inhomogeneous wrist model described below. The VHP-Female model characterizes a 60 year old Caucasian female subject with a height of 162 cm as measured from top of the scalp to the average center of both heels. The body mass of the model is 88 kg, resulted in a computed Body Mass Index of 33.5 (moderately obese). The model has separate anatomical skin and fat layers of variable thicknesses and has been augmented with electromagnetic tissue properties from the IT’IS Database^42^ in the frequency range from 10 MHz to 100 GHz.

**Figure 4:**
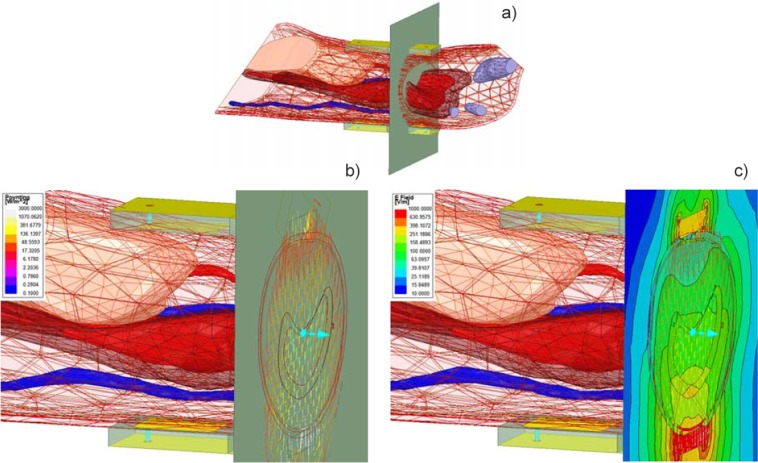
(**a**) Computational model configuration. The RX dual patch antenna is on top of the wrist; the TX antenna is on its bottom. (**b**) Distribution of the Poynting vector through the wrist cross-section with the lower threshold of 0.1 W/m^2^ and the upper threshold of 3000 W/m^2^ or 0.3 W/cm^2^. The vast majority of the radiated power propagates through the center of the wrist and through the bone marrow toward the receiver antenna while a vanishingly small power flow is observed close to the perimeter of the wrist. (**c**) Distribution of the complex magnitude of the total electric field through the wrist cross-section.

A wrist model from the VHP-Female with 10 individual tissue sub-compartments has been isolated, augmented with the antenna models from Fig. 1a, and simulated at 915 MHz using the FEM based software ANSYS HFSS Electronics Desktop 2019R1 with seven adaptive mesh refinement passes, and a given input power of 1 W into each radiator of the dual antiphase patch antenna on bottom of the wrist, mimicking the transmitter (TX) setup. The receiver (RX) arrangement includes an identical dual antiphase patch antenna on top of the wrist. The model configuration is shown in Fig. 4a.

Simulation results are shown in Fig. 4b,c, respectively. Figure 4b demonstrates distribution of the Poynting vector across the wrist cross-section with the lower threshold of 0.1 W/m^2^. Simulation results reveal that the vast majority of the radiated power propagates through the center of the wrist and through the bone marrow toward the receiver antenna. A vanishingly small power flow is observed close to the perimeter of the wrist. This is a consequence of the dual antiphase patch antenna design described in the previous section. The effects of both wave diffraction and of the associated surface waves around the wrist thus appear to be negligibly small, as seen in Fig. 4b. Additionally, Fig. 4c shows the distribution of the complex magnitude of the total electric field through the wrist cross-section with the most significant transmitted field observed for cortical and trabecular bone. Similar results have been obtained at 600 MHz and 1200 MHz, respectively.

This simulation model does not take into account anisotropy of trabecular bone since we were unable to find the anisotropic dielectric material properties for the given frequency bands. We also mention the lack of data on dielectric radiofrequency properties of the osteoporotic bone in the literature. Trabecular or cancellous bone forms the inner part of the medullary cavity in short and flat bones. In trabecular bone, the anisotropic calcified tissue is arranged in the form of plates or struts called trabeculae, approximately 200 µm thick, creating numerous interconnected cavities^71^. These cavities are filled with bone marrow. In osteopenic/osteoporotic bone, the trabecular bone matrix is partially replaced by a soft fatty tissue. Correlation of mechanical anisotropy with dielectric bone properties has been discussed in ref. ^72^. Also note that the isotropic dielectric data given in ref. ^42^ were obtained with animals *in vivo* and *in vitro*, and therefore could hardly be considered as entirely accurate for the human wrist. For instance, ref. ^72^ reports rather different values of the dielectric constant for human trabecular bone. Additional relevant research on bone dielectric properties was performed in refs. ^38,72–74^.

### Device construction and measurement sequence

**Figure 5:**
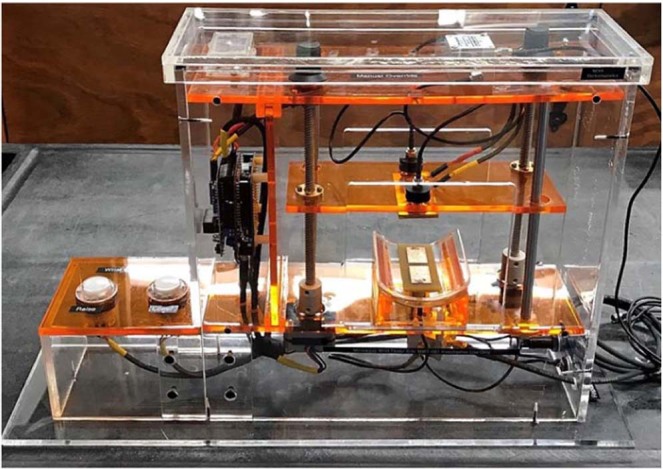
Microwave setup with transmitting/receiving dual antiphase patch antennas (each comprising of two closely spaced patch antennas fed in antiphase), gear motors, pressure sensors, and a microcontroller.

A prototype for the radiofrequency wrist tester is shown in Fig. 5. It includes a transparent plastic enclosure, a movable top frame (using two stepper motors with a microcontroller connected to the pressure sensors), two 2×1 antenna arrays described previously, and four pressure sensors. Wrist measurements are performed when a controlled pressure of 1 kg of force is applied. We substantially increase the measurement repeatability and accuracy of the device by using a pressure-controlled, solid, and accurately adjustable wrist support. This allows us to precisely control pressure during antenna attachment to the wrist. The testbed in Fig. 5 includes the following major components:

- Movable top with four pressure sensors (FlexiForce^®^ ESS301) connected to a microcontroller and a 2 × 1 receiver antenna array with a 180° power splitter (ZFSCJ-2-4-S, Mini-Circuits). The pressure sensors and the array use a flexible support to enable better adjustment to various wrist sizes;
- Fixed bottom with the identical embedded (and replaceable) 2 × 1 transmit antenna array printed on 128 mil FR4 and another 180° power splitter; two cables from the splitter are wired together;
- Supporting frame, which could potentially measure wrist thickness after applying pressure from top;
- A grip (not shown in Fig. 5) to fix the arm position.

Measurements are performed by putting the wrist inside the device holder horizontally at a prescribed position of the ulnar head. The device top moves down and stops automatically when the applied pressure reaches 1 kg of force (3–7 sec), irrespective of wrist diameter. Measurements of reflection and transmission coefficients ${S}_{11}(f)$, ${S}_{21}(f)$ after the 180 deg power splitters as functions of frequency are performed in less than 0.5 seconds. The device top is then raised to its original position in 3–7 sec. The entire measurement sequence requires about 20–30 sec. Left and right wrist circumferences are separately measured and recorded at a position just under the ulnar head.

Electronics and patient safety are addressed by both the low power of the system and the construction therein. All wiring connections are sealed and all exposed electronics are isolated. We use two clearly labeled replaceable external fuses (7 V 3 A).

From the point of view of electromagnetic safety, the present device is equivalent to two low-power cellphones placed on either side of the wrist: one is transmitting and another is receiving. The device introduces minimum radiofrequency energy into the wrist (0.1 W total power over a maximum time duration of 0.5 min). Such power is 6–30 times less than the power of a cellphone and is 10,000–100,000 times less than the power of an MRI radio-frequency coil. The U. S. Department of Health and Human Services, Food and Drug Administration, Center for Devices and Radiological Health, Division of Biomedical Physics accepts guidelines of the International Commission on Non-Ionizing Radiation Protection (ICNIRP)^75^. These guidelines state that the general-exposure local Specific Absorption Rate (SAR) should be less than 0.08 W/kg at 915 MHz^75^, which is the center operating frequency of the device. The corresponding numerical simulation study performed with the commercial FEM software ANSYS Electronics Desktop 2019R1 and a CAD VHP-Female version 3.0 full-body computational human model^65^ revealed that this condition is satisfied with a 10x safety margin.

Finally maintaining the cleanliness of the patient contact areas on the device is addressed by the use of thin long-sleeve gloves for each patient, preventing any direct physical contact with the device; and the device is periodically cleaned with alcohol swabs.

**Figure 6:**
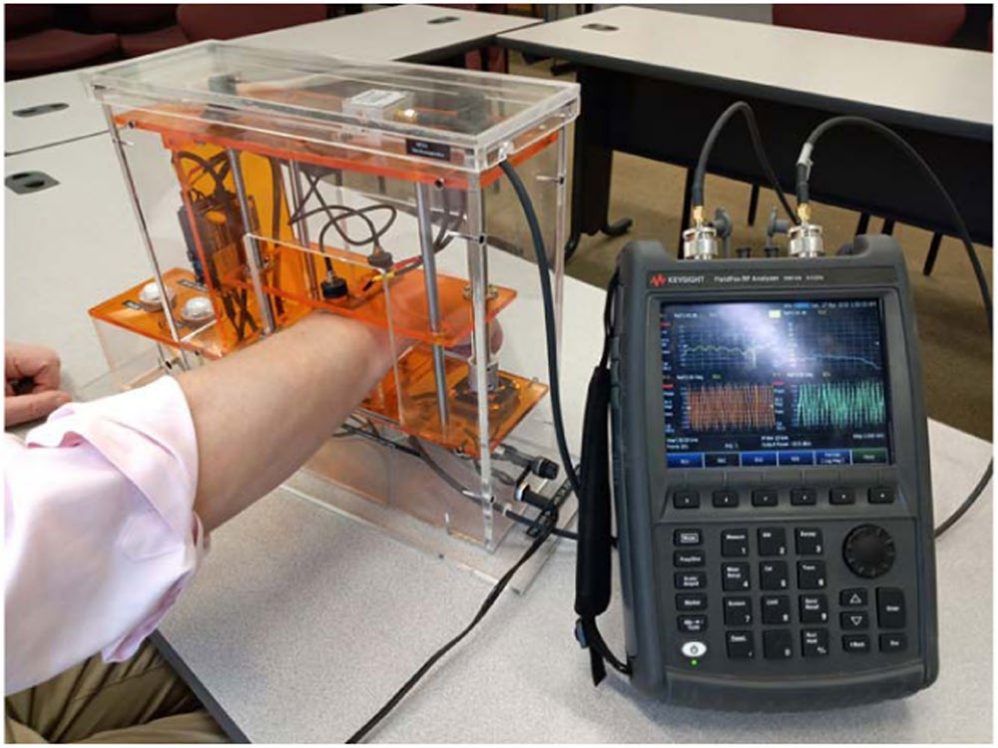
Experimental setup connected to portable Keysight FieldFox N9914A network analyzer and performing measurements. Measurements of reflection and transmission coefficients ${S}_{11}(f)$, ${S}_{21}(f)$ as functions of frequency are performed in less than 0.5 seconds. The device top is then raised to its original position (3–7 sec).

In the research study reported below, the antennas have been connected to a portable Keysight FieldFox N9914A network analyzer in order to investigate and test the entire frequency band from approximately 0 (precisely 30 kHz) to 2 GHz and to establish the most sensitive region(s) of operation. The typical operation setup is shown in Fig. 6.

### De-embedding transmission and reflection coefficients

Since the radiofrequency device is contained in a protective enclosure, the S-parameters cannot be calibrated and measured directly at the power splitter ports. The microwave device was therefore calibrated along with the cables running to the network analyzer. This leads to oscillatory behavior of both ${S}_{11}(f)$ and ${S}_{21}(f)$. To eliminate these spurious oscillations, de-embedding was performed after measuring S-parameters for each cable. For this purpose, the MATLAB RF Toolbox™ from MathWorks, Inc. was employed (function deembedsparams).

## Results

### Pilot study

After receiving Institutional Review Board (IRB) approval through Worcester Polytechnic Institute, the written informed consent from 72 subjects (ranging from 23–94 years old, 58 female, 14 male, 3 African American, 4 Hispanic, 64 Caucasian, cf. Appendix B) was obtained to participate in this study. All measurements were further performed in accordance with the relevant IRB guidelines and regulations.

Given a common fear of ionizing radiation, especially among elderly subjects, we did not enforce or require any extra DXA measurements. For every subject the following parameters were recorded:

- Reflection coefficient ${S}_{11}(f)$ (both magnitude and phase) from right and left wrists for the bottom antenna configuration after the 180 deg power splitter in the frequency range 30 kHz-2 GHz;
- Transmission coefficient ${S}_{21}(f)$ (both magnitude and phase) between the two antennas after the 180 deg power splitters and through right and left wrists, respectively, in the frequency range 30 kHz-2 GHz;
- Age, weight, height, left wrist circumference, and right wrist circumference;
- Family history of osteoporosis and history of bone fracture according to a verbal statement.

Initially, all subjects with de-identified data have been subdivided into five preliminary categories detailed in Appendix B:

1. **Category 1** (**healthy young adults**, 23–30 years old). Unknown bone density but young age (≤30). **5 subjects in total** (2 female, 3 male);
2. **Category 2** (**low risk factor category**, 42–94 years old). Unknown bone density (no DXA data) but (all together): no history of bone fractures, no medication, and no family history of bone fracture/osteoporosis. **32 subjects in total** (24 female, 8 male). Emphasize that these clinical risk factors can have a larger impact on fracture risk than one standard deviation decline in bone density^76,77^. Therefore, we feel comfortable considering them at low risk without explicit BMD information;
3. **Category 3 (unknown risk factor category**, 44–77 years old**)**. Unknown bone density (no DXA data) but at least one of the following: family history of osteoporosis, low BMI, history of bone fractures, women after menopause. **12 subjects in total** (10 female, 2 male);
4. **Category 4 (osteopenia or low bone density**, 55–90 years old**)**. Confirmed osteopenic bone density (T-score between – 1.0 and –2.4) according to the most recent DXA exam (obtained within the last year) and prescribed medications such as various calcium/magnesium supplements (600–1000 mg). **18 subjects in total** (17 female, 1 male);
5. **Category 5 (osteoporosis**, 55–86 years old**)**. Confirmed osteoporotic bone density (T-score of –2.5 or below) according to the most recent DXA exam (obtained within the last year) and prescribed medications such as bisphosphonates. **5 Subjects in total** (5 female, 0 male).

### Subject selection for dichotomous diagnostic test (Osteogenic/Osteoporotic vs Healthy)

For the unbiased study, we eliminated Category 3 (unknown risk factor category). As a result, we ended up with two groups (60 subjects in total) suitable for binary classification:

1. **Group 1 osteopenic/osteoporotic** (Categories 4 and 5 together, 55–90 years old, mean 77.5/STD 10.1). T-score of –1.0 or below according to the most recent DXA exam and prescribed medications. **23 subjects in total** (22 female, 1 male).
2. **Group 2 healthy** (Categories 1 and 2 together: low risk category, 23–94 years old, mean 60.2/STD 16.6). Unknown bone density (no DXA data) but young adults or (all together): no history of bone fractures, no medication, and no family history of osteoporosis. **37 subjects in total** (26 female, 11 male).

A single binary statistic (the Youden’s J statistic) was then applied to these two groups to capture the performance of a dichotomous diagnostic test.

### Data processing for entire frequency band – transmission through the wrist

**Figure 7:**
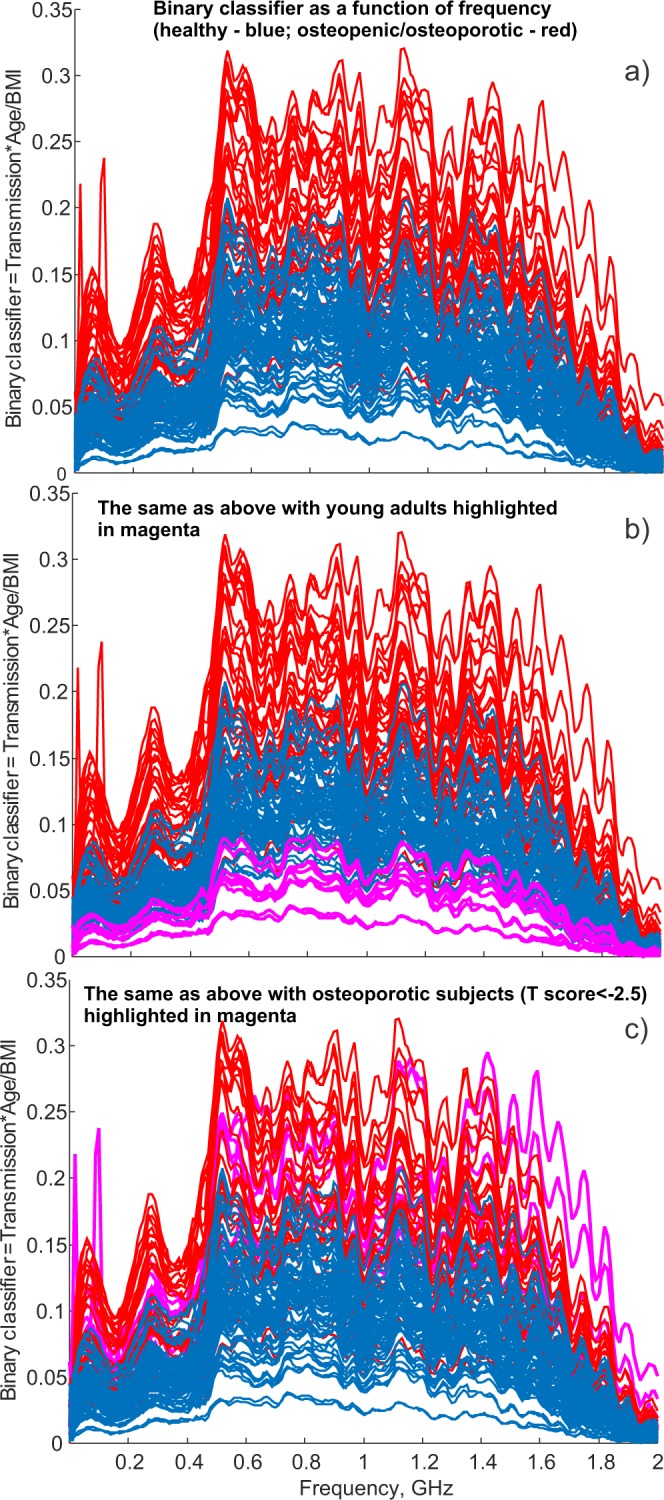
(**a**) Transmission coefficient $|{S}_{21}(f)|$ between the two antennas and through left and right wrists divided by BMI and multiplied by age in the frequency range 0–2 GHz for all subjects from Group 1 (osteopenic/osteoporotic) and Group 2 (healthy). Red color corresponds to Group 1 while blue color corresponds to Group 1. 120 frequency curves in total are shown in the figure. (**b**) The same as in a) but with the data for five young adults highlighted in magenta. (**c**) The same as in (**a**) but with the data for five osteoporotic subjects (T score below – 2.5) highlighted in magenta.

Figure 7a shows the normalized transmission coefficient magnitude between the two antennas through left and right wrists in the 0–2 GHz frequency band for all 60 subjects selected for the dichotomous diagnostic test as described previously. Normalization means that we divide $$|{S}_{21}(f)|$$ by BMI and multiply it by subject age, that is $$Age\cdot |{S}_{21}(f)|$$$$/{\rm{BMI}}$$. Red color corresponds to Group 1 while blue color corresponds to Group 2. One hundred and twenty frequency curves in total are shown in the figure.

One observes that the osteoporotic and osteopenic subjects more consistently indicate higher normalized transmission coefficients, while the healthy subjects more consistently indicate lower normalized transmission coefficients. In other words, the osteoporotic and osteopenic wrists become relatively more transparent to radio frequency signals. However, some curves in Fig. 7 locally overlap. An integral measure of the transmission coefficient may therefore be the best differentiator. Notably, the data for five young healthy subjects highlighted in magenta in Fig. 7b indicate the smallest “transparency” while the data for five osteoporotic subjects (T-score of –2.5 or below) highlighted in magenta in Fig. 7c indicate the nearly highest “transparency”.

### Index functions

Bone density presumably correlates with BMI and approximately inversely with age; osteoporosis mostly occurs in elderly women with a low BMI. Since our cohort mostly includes women, we could neglect the sex variable. Thus, for comparison purposes, we will introduce a simple “natural” indicator, ${D}_{0}$, of osteoporosis/osteopenia for the study participants in the form of the ratio of two clinical risk factors:

1 $${D}_{0}=\frac{Age}{BMI}$$

which does not require any measurements but should already approximately differentiate osteoporotic/osteopenic and healthy persons, respectively. Healthy conditions correspond to lower values of ${D}_{0}$. Indictor ${D}_{0}$ will be normalized to its maximum value, that is

2 $${D}_{0}\to {D}_{0}/\,{\rm{\max }}({D}_{0})$$

Now, we introduce the primary differentiator (index) in the form

3 $${D}_{1}=\frac{Age}{BMI}\times {\int }_{{f}_{L}}^{{f}_{U}}|{S}_{21}(f)|df$$

which additionally includes an integral of the transmission coefficient ${S}_{21}(f)$ from Fig. 3 over a certain frequency band from ${f}_{L}$ to ${f}_{U}$. Indicator ${D}_{1}$ is obtained by averaging the data for both wrists. Indictor ${D}_{1}$ is also normalized to its maximum value as

4 $${D}_{1}\to {D}_{1}/\,{\rm{\max }}({D}_{1})$$

Following the observations of Fig. 7, we suggest that indicator ${D}_{1}$ would better differentiate osteoporotic/osteopenic and healthy conditions, respectively, than indicator ${D}_{0}$.

### Data processing for a narrow frequency band of 0.1 *GHz*

Using the network analyzer for RF data acquisition and obtaining the entire spectrum in Fig. 7 is costly. Low-cost power meters with the center frequency from 0.5 to 1 GHz operating over the band of 0.1 GHz could be designed and/or purchased. Therefore, we restrict ourselves to a smaller frequency band in Eq. (3). We assume that the integration bandwidth is given by

5 $${f}_{U}-{f}_{L}=0.1\,\text{GHz}$$

**Figure 8:**
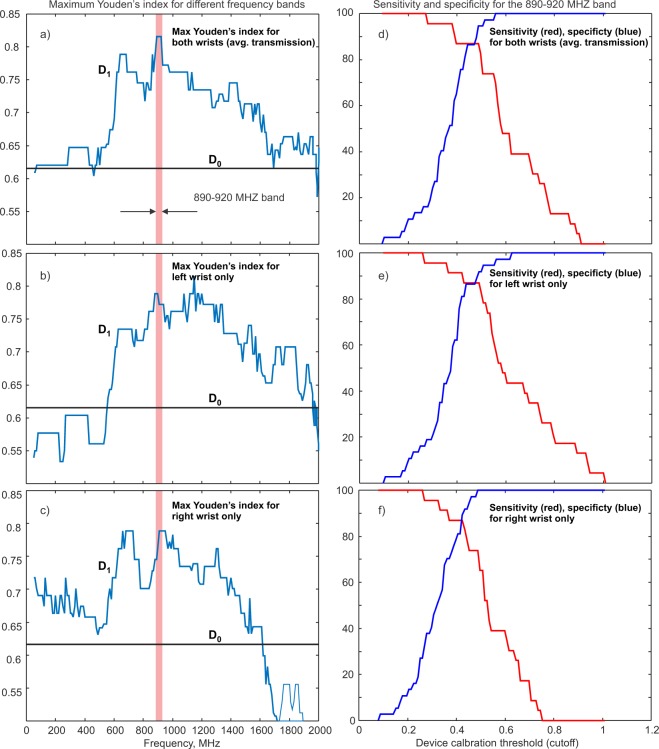
(**a**) Maximum Youden’s J index for every center frequency of the moving 0.1 GHz band using the index given by Eq. (3). Average transmission coefficient for both wrists is used. (**b**) The same as in a) but only the transmission coefficient for the left wrist is used. (**c**) The same as in a) but only the transmission coefficient for right wrist is used. The most favorable band position indicated by a vertical bar is from 890 to 920 MHz. The straight horizontal line in (**a–c**) shows Youden’s J index for the simple indicator ${D}_{0}$ given by Eq. (1). (**d**) Sensitivity (red) and specificity (blue) corresponding to the band 890–920 MHz in (**a**). Both are given as functions of the device threshold value $T$ in Eq. (6). (**e**,**f**) the same as in d) but only the transmission coefficient for the left or right wrist, respectively, is used.

We chose the center frequency of the band in such a way as to provide the best performance. To do so, we move the integration window with the width of $0.1\,{\rm{GHz}}$ in Fig. 7 from left to right. Then, we select such window positions where the ROC area and Youden’s J index for the ROC (receiver operating characteristic) curve are maximized as shown in Fig. 8.

### Youden’s J index for Eq. (3). Finding optimum frequency band

Figure 8a shows the maximum value of Youden’s J index for each center frequency of the band in Eq. (3). Here, an average absolute transmission coefficient $|{S}_{21}(f)|$ for both wrists of the subject is employed. The maximum value of Youden’s J index corresponds to the optimum value of the empirical device calibration threshold or cutoff $T$. Threshold $T$ differentiates the two states:

6a $$Osteopenic/Osteoporotic\,{D}_{1} > T$$

6b $$Healthy\,{D}_{1}\le T$$

The most favorable frequency band indicated by a vertical bar in Fig. 8 -left is from 890 to 920 MHz. The horizontal line in Fig. 8a–c shows Youden’s J index for indicator ${D}_{0}$ given by Eq. (1). The global maximum value of Youden’s J index for indicator ${D}_{1}$ achieved over the band from 890 to 920 MHz is 0.815 or 81.5%. Emphasize that the global maximum value of Youden’s J index for indicator ${D}_{0}$ is 0.615 or 61.5%. The difference in these values reflects supplementary useful information obtained from radiofrequency data.

Figure 8b shows the same result as in Fig. 8a but when only the transmission coefficient for the left wrist of every subject is used. Similarly, Fig. 8c uses the data for the right wrist only. The information from both wrists is more beneficial than the information obtained from a single wrist.

### Sensitivity and specificity for the optimum frequency band

Both sensitivity (red) and specificity (blue) are shown in Fig. 8d at the band center frequency of ~900 MHz corresponding to the global maximum of Youden’s J index in Fig. 8a. The *x*-axis is the device calibration threshold value $T$ in Eqs. (6). One observes that we simultaneously reach the sensitivity of 87% and the specificity of 87% or better when the device is calibrated with the threshold value $0.44\le T\le 0.49$ in Eqs. (6). This corresponds to the calibration threshold tolerance of ±3%. Figure 8e shows the same result as in Fig. 8d but when only the transmission coefficient for the left wrist of every subject is used; Fig. 8f uses the data for the right wrist only. The information from both wrists is evidently more beneficial than the information obtained from one single wrist.

For the present binary classifier, sensitivity for five severely osteoporotic subjects (T-score of −2.5 or below, indicated with magenta color in Fig. 7c) is 100%. Similarly, specificity for five young healthy adults (indicated with magenta color in Fig. 7b) is also 100%.

## Discussion

### Improvement of antenna matching

The simplified numerical model of the human wrist used in antenna optimization does not take into account realistic wrist composition, variations in tissue properties at different frequencies or tissue anisotropy. Therefore, we were unable to properly match antennas over the entire targeted frequency band from approximately 200 MHz to 2 GHz. Instead, an “average” matching to $10-j5\,{\rm{ohm}}$ was used. This is not entirely adequate (as shown by measured ${S}_{11}$ data in Appendix A) and is a subject of possible improvement.

Matching an on-body antenna coupled with a power splitter (beamformer) over the wide frequency band (~200 MHz – 2 GHz or 10:1 in the present case) is a difficult task. Instead of performing further numerical simulations, we plan on using the already available measured ${S}_{11}$ data for 60 (Appendix A) or 72 (the entire study pool) subjects and constructing a new matching network, which is directly based on the experimental ${S}_{11}$ data obtained in this study.

### Effect of surrounding tissue

As indicated already in Fig. 1a, the device measures an integral estimate of radio frequency propagation through the wrist. This integral estimate involves not only bone but also cartilage and soft tissues surrounding bone. And yet, this estimate correlates quite well with both the clinical risk factors and the subsequent DXA data as shown in the present study. This is likely due to the fact that a significant portion of the radio frequency signal path in Fig. 1a still passes through trabecular bone as demonstrated in Fig. 4.

Another point is the small thickness of the fat layer around the wrist, perhaps the smallest when compared with other body regions. This small fat layer thickness does not support multipath (signal propagation along the fat layer^78^ instead of through the bone). Furthermore, the wrist composition is less significantly affected by age. In Ref. ^79^, skin thickness and subcutaneous fat thickness were measured on the back of the hand near the wrist between the second and third metacarpal and above the third metacarpal bone. The average values for young adults were 0.5 and 0.6 mm, respectively. With aging, the average skin thickness did not change significantly while the average subcutaneous fat thickness decreased by approximately 30% (from 0.6 to 0.4 mm)^79^. This is in contrast to other parts of the body where fat accumulation with age may be very significant^80^. Finally, an additional factor might involve accompanying changes in cartilage properties and muscular tissue properties which may be specific for the osteopenic/osteoporotic subjects.

### Effect of wave diffraction around the wrist, surface waves

The effects of both wave diffraction and the associated surface waves around the wrist have been found to be small for the present antenna setup at 900 MHz as well as at 600 and 1200 MHz. The corresponding simulation results shown in particular in Fig. 4 reveal that, for the dual antiphase patch antenna setup, the bulk of the radiated power propagates through the center of the wrist and the bone marrow toward the receiver antenna while a vanishingly small power flow is observed close to the perimeter of the wrist.

The ripples in Fig. 7 are due to non-adequate antenna matching over the entire frequency band for a variety of different subjects. The proof of this is given in Fig. A1 of Appendix A where the ripples of S21 correlate with the ripples of S11 (a local minimum of S11 corresponds to a local maximum of S21) all the way up to at least 1 GHz.

An antenna backlobe observed in Fig. 4c has been found to provide a minimum multipath effect that is below noise floor. The proof uses experiments with the RX antenna somewhat lifted up above the wrist. In every tested case, the corresponding transmission coefficient was below −50 dB over the entire band. This value was selected as the noise floor. Only when the TX antenna was pressed against the wrist were the reported transmission coefficients on the order of –20 to –40 dB observed.

### Results for male and female subjects

The selected study groups were composed primarily of women (representative of the patient population that gets osteoporosis). However, results for one osteoporotic male subject and eleven control male subjects were also compiled. The separate results for the two genders are as follows:

- For female subjects: sensitivity of 86% and the specificity of 85% when the device is calibrated with the threshold value $0.44\le T\le 0.49$ in Eqs. (6).
- For male subjects: sensitivity 100% and specificity of 90% when the device is calibrated with the threshold value $0.44\le T\le 0.49$ in Eqs. (6).

### Results for healthy young subjects

We have intentionally included the young healthy adults into control to demonstrate a 100% specificity for this subgroup. If we were to exclude them, nearly the same results for sensitivity and specificity (approximately 87%) would be obtained. However, the control group will now have a higher mean age of 65.3 with STD of 10.9.

### Radio frequency data add supplementary useful information to clinical risk factors

The index function used in this study is not the radiofrequency transmission coefficient alone, but the transmission coefficient multiplied by age and divided by BMI. The inclusion of these two clinical risk factors increases both sensitivity and specificity. At the same time, the inclusion of radiofrequency transmission data adds useful supplementary information to the clinical risk factors. This is best seen in Fig. 8 where such an inclusion increases the Youden’s index by 20% (from 61.5% to 81.5%) when the frequency band from 890 to 920 MHz is considered. Non-normalized data for the sole transmission coefficient are given in Appendix A.

### How much supplementary information is contained in “effective” wrist thickness

The index function used in this study does not involve the wrist thickness or its circumference. On the other hand, the transmission coefficient clearly increases for a thinner (more likely osteopenic/osteoporotic) wrist and decreases for a thicker (more likely healthy) wrist. One may therefore suggest that the bulk of added radiofrequency information is simply the “effective” (from the viewpoint of radiofrequency propagation) overall wrist thickness, which may be considered as another added clinical risk factor.

**Figure 9:**
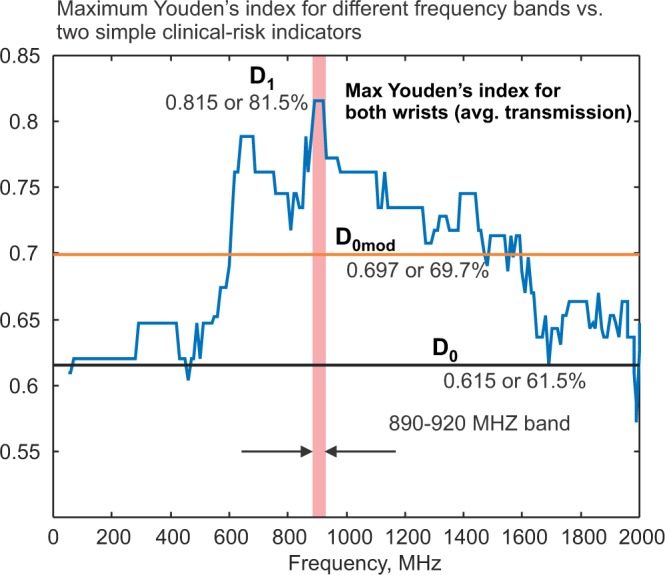
Blue - maximum Youden’s J index for every center frequency of the moving 0.1 GHz band using the index given by Eq. (3) with radiofrequency measurements. Average transmission coefficient for both wrists is used. Straight horizontal lines show Youden’s J index for the simple indicator ${D}_{0}$ given by Eq. (1) and its modification ${D}_{0{\rm{mod}}}$ from Eq. (7).

While this is partially true, the radiofrequency measurements still provide more useful information than the simple mechanical measurement of the wrist thickness or its circumference. The first proof is given in Fig. 9. This is a replica of Fig. 8a where we additionally plot the Youden’s index for the natural indicator ${D}_{0}$ from Eq. (1) modified by the mechanical measurements of the wrist circumference (mean of both wrists was taken), which were performed for ever subject, that is

7 $${D}_{0{\rm{mod}}}=\frac{Age}{BMI\cdot wrist\,circumference}$$

One observes that the performance of ${D}_{0}$ improves but the radiofrequency data remain more informative in the frequency band from 600 to 1600 MHz where the antenna matching is the best.

The second proof is given in Appendix A where we present in Fig. A3 the non-normalized data for the sole linear transmission coefficient but multiplied by the wrist thickness (more precisely – by the wrist circumference). This is done to “undo” the effect of the mechanical thickness, assuming approximately linear with distance radiofrequency damping. The differentiation between healthy and osteopenic/osteoporotic groups becomes poorer, but it is still in place.

### Open problems

Other questions and problems which require further investigation include:

- Using all measured information. Three fourths of the measured information have not been used for data processing in the present study. The non-processed data include phase information for both transmission and reflection coefficients and the amplitude information for the reflection coefficient. Our study indicates that some classifiable phase variations may be observed for the phase of the transmission coefficient. More advanced deep learning algorithms might perhaps help to solve this problem.
- Construction of a low-cost device. The present setup uses a high-cost professional network analyzer. After selecting the proper frequency band and restricting to amplitude measurements, a low-cost setup on the basis of a narrowband microwave power meter could be constructed. This is a relatively simple technical task.

## Conclusions

In this study, a potentially low-cost, through-transmission radiofrequency device to detect low bone density conditions was designed, constructed, and tested. The device uses novel on-body antennas (dual antiphase patch antennas) connected to both sides of the human wrist under controlled applied pressure. It was observed that osteopenic and osteoporotic subjects more consistently indicate higher normalized transmission coefficients, while reduced risk subjects more consistently indicate lower normalized transmission coefficients, either normalized or not.

A pilot study with 72 subjects has been performed. For the dichotomous diagnostic test, we have selected 60 study participants (23–94 years old, 48 female, 12 male) who could be positively differentiated between the osteopenic/osteoporotic and healthy complementary states, respectively. The osteopenic state was determined based on a DXA T-score between –1 and –2.5; and the osteoporotic state was determined based on a DXA T-score below –2.5. No DXA measurements have been performed for healthy subjects but all subjects from this category passed all of the following clinical risk-factor tests: no history of bone fractures, no medication, and no family history of bone fracture/osteoporosis The band-limited integral of the transmission coefficient averaged for both wrists multiplied by age and divided by BMI has been used as an index.

Youden’s J statistic was applied for center band frequencies in the range from 890 to 920 MHz. For a 100 MHz wide frequency band, the maximum Youden’s J index is 81.5%. Both the sensitivity and specificity simultaneously reach 87% given the calibration device threshold tolerance of ±3%. At the same time, sensitivity for severely osteoporotic subjects (DXA T-score of −2.5 or below) is 100% while specificity for young healthy adults is also 100%.

In conclusion, our approach correlates well with the available DXA measurements and has the potential for screening patients at risk for fragility fractures, given the ease of implementation and low costs associated with both the technique and the equipment. The inclusion of radiofrequency transmission data adds significant supplementary information to the available clinical risk factors.

## Supplementary information

Supplementary information.

## Data Availability

All raw measurement data for 72 subjects along with data description and custom MATLAB processing scripts used in this study are made available through NIH Figshare Database through the following reference: Dataset for “Concept of a Radiofrequency Device for Osteopenia/Osteoporosis Screening”. 03.02.2020. *NIH Figshare*. 10.35092/yhjc.11783697.v1. The equivalent link: https://nih.figshare.com/articles/Dataset_for_Concept_of_a_Radiofrequency_Device_for_Osteopenia_Osteoporosis_Screening_/11783697/1.
