# Supplementary material for: Concept of a Radiofrequency Device for Osteopenia/Osteoporosis Screening
Source: Sci Rep. 2020 Feb 26;10:3540. doi: 10.1038/s41598-020-60173-5 (PMC7044313; doi:10.1038/s41598-020-60173-5)
Supplement: Supplementary file 1 — Supplementary information. [file 41598_2020_60173_MOESM1_ESM.pdf]

Concept of a Radiofrequency Device for Osteopenia/Osteoporosis Screening

Sergey N. Makarov*[1-3], Gregory M. Noetscher[1,3], Seth Arum[4], Robert Rabiner[5], and Ara Nazarian[6]

[1]ECE Dept., Worcester Polytechnic Institute, Worcester, MA 01609, USA
[2]Athinoula A. Martinos Center for Biomedical Imaging, Massachusetts General Hospital, Harvard Medical School, Boston, MA 02114, USA
[3]Neva Electromagnetics, LLC., Yarmouth Port, MA 02675, USA
[4]Alnylam Pharmaceuticals, Cambridge, MA 02412, USA
[5]IlluminOss Medical, East Providence, RI 02914, USA
[6]Center for Advanced Orthopaedic Studies, Beth Israel Deaconess Medical Center, Harvard Medical School, Boston, MA 02215

Supplementary Information

APPENDIX A. NON-NORMALIZED SCATTERING DATA

APPENDIX B. STUDY SUBJECT ROSTER

APPENDIX A. NON-NORMALIZED SCATTERING DATA

Below, we report the complete non-normalized scattering data (i.e., without division by BMI and multiplication by age) for all subjects selected for dichotomic diagnostic binary test in Section III, including the discrete data for each wrist and separate magnitude and phase data for both scattering coefficients (reflection and transmission). We also report magnitude of the non-normalized transmission coefficient multiplied by wrist circumference as suggested in the man text (Discussion section).

A. Reflection and transmission coefficients for left and right wrists

Fig. A1 shows non-normalized reflection (red) and transmission (blue) coefficients in decibel for a typical osteoporotic subject from Group 1 (Fig. A1a) and a typical healthy subject from Group 2 (Fig. A1b). While the reflection coefficients for both wrists are barely distinguishable, the transmission coefficients may show a more significant deviation. For the right wrist, the transmission coefficient is typically somewhat lower.

Note that the non-normalized transmission coefficients for Group 1 are higher than transmission coefficients from Group 2 by 3– 6 dB in average. These numbers correspond to the received power ratios from 2 to 4. Also note the noise floor for the present device configuration is approximately –50 dB and the dynamic range for the transmission coefficient is approximately 30 dB,

B. Magnitude and phase of non-normalized scattering coefficients

Fig. A2 shows magnitude and phase of transmission coefficient $S_{21}(f)$ and reflection coefficient $S_{11}(f)$. A linear scale is used for the magnitude and the scale in degrees for the phase. Subjects from Group 1 (osteopenic/osteoporotic) are again marked red and subjects from Group 2 (healthy control) are marked blue. Even without normalization, the transmission coefficient already differentiates between the two groups rather well but certainly not as well as with the extra information involving subjects' BMI and age. On the other hand, the reflection coefficient magnitude (Fig. A2c) hardly indicates any differentiation between the two groups; the same may said of reflection coefficient phase (Fig. A2d). This observation is to be expected.

Interestingly, some additional useful information may be contained in the phase of the transmission coefficient shown in Fig. A2b. Healthy wrists (shown in blue) more consistently indicate a steeper phase ramp with increasing frequency. This information has not yet been processed.

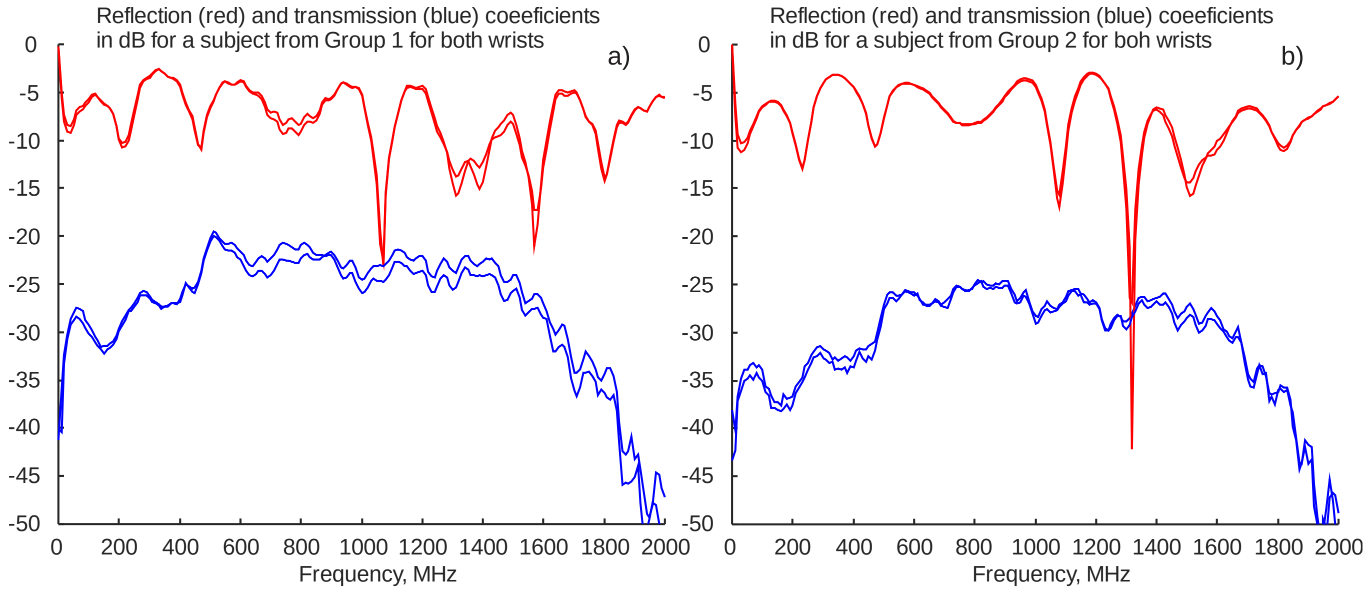

Fig. A1. a) – Reflection (red) and transmission (blue) coefficients in decibel for a typical osteoporotic subject (Group 1) for every wrist separately. b) – The same coefficients for a typical healthy subject (Group 2). The right wrist typically indicates a lower transmission coefficient.

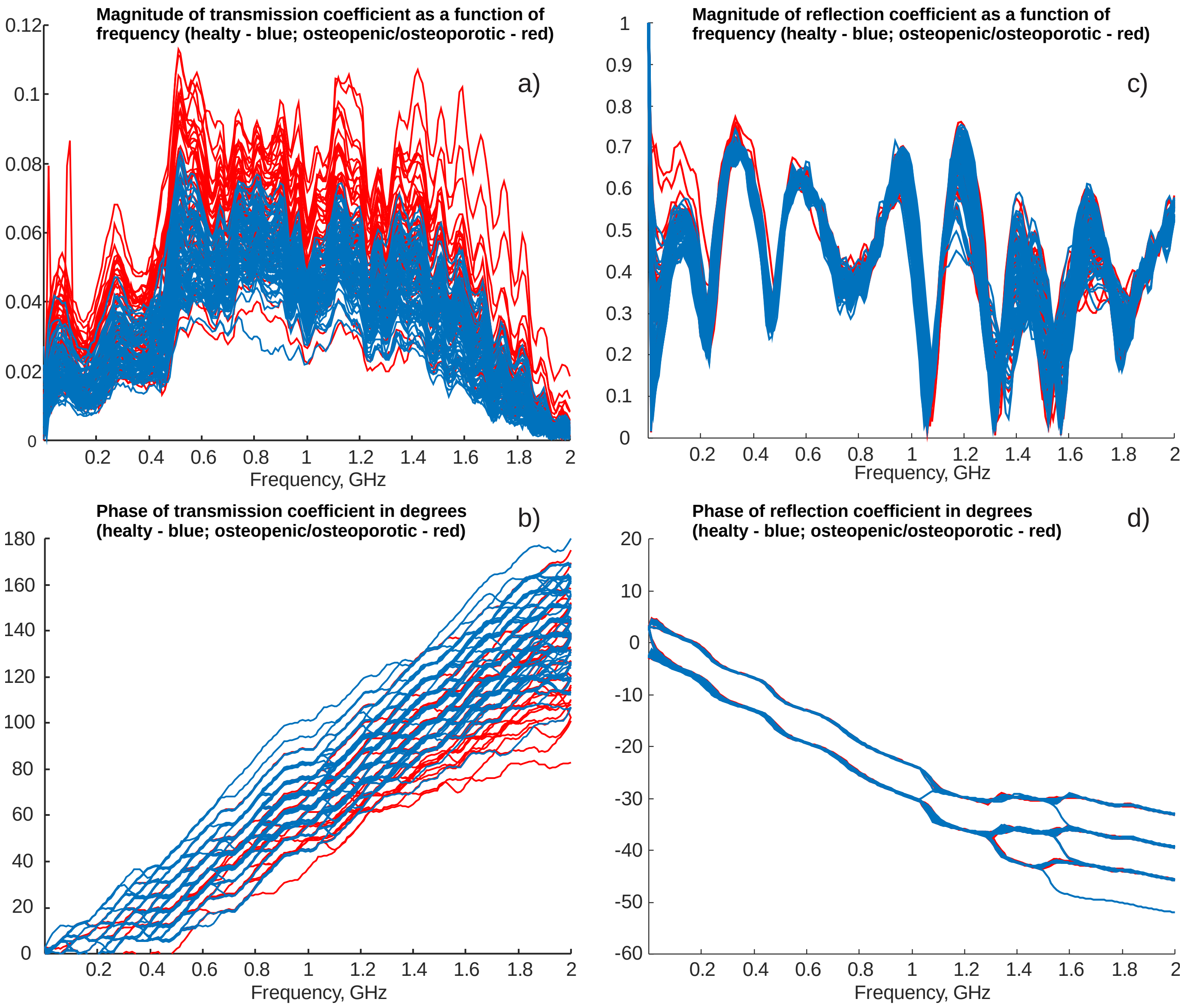

Fig. A2. Magnitude (a) and phase (b) of non-normalized transmission coefficient $S_{21}(f)$ and non-normalized reflection coefficient $S_{11}(f)$ in (c) and (d). A linear scale is used for the magnitude and the scale in degrees for the phase. Subjects from Group 1 (osteopenic/osteoporotic) are again marked red and subjects from Group 2 (healthy control) are marked blue. Total 120 curves are shown in every plot.

C. How much supplementary information is contained in effective wrist thickness?

We present in Fig. A3 the non-normalized data for the linear transmission coefficient but multiplied by the wrist thickness (more precisely – by the wrist circumference averaged for both wrists and divided by the maximum wrist circumference of the entire dataset). The wrist circumferences were measured for every subject for the dataset.

The multiplication is done to “undo” the effect of the mechanical thickness, assuming approximately linear with distance radiofrequency damping. The differentiation between healthy and osteopenic/osteoporotic groups in Fig. A3 becomes poorer than in Fig. A2a. This means that the radiofrequency differentiation partially reflects the wrist thickness, indeed. However, the overall differentiation in Fig. A3 is still in place. Its main reason is likely the sole effect of the tissue properties themselves, including bone and surrounding tissues. This effect is rather substantial in the present study; it leads to the increase of Youden's J index from 69.7% to 81.5% in Fig. 9 of the main text.

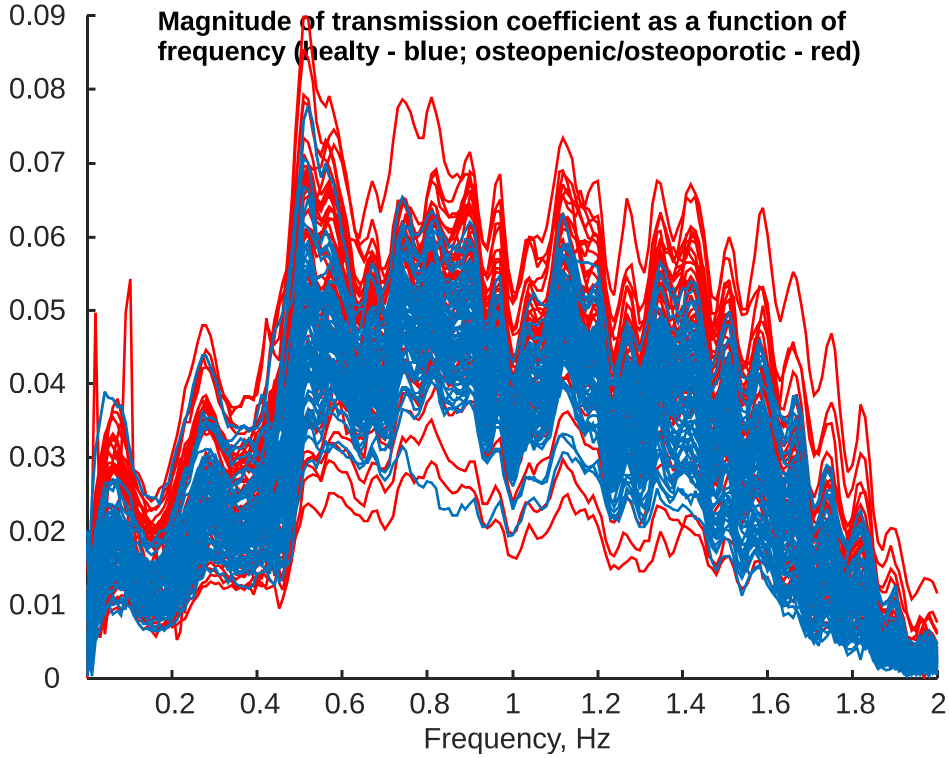

Fig. A3. Magnitude of non-normalized transmission coefficient $S_{21}(f)$ multiplied by wrist circumference (and normalized by maximum circumference). Subjects from Group 1 (osteopenic/osteoporotic) are marked red and subjects from Group 2 (healthy control) are marked blue. Total 120 curves are shown in the plot.

APPENDIX B. STUDY SUBJECT ROSTER

The study subject roster is given in Table B1 below.

Table B1. Study roster (from left to right): subject number, category score (1-5), age, weight, height, sex, left wrist circumference, and right wrist circumference.

Subject #	Osteo Category	Age	Weight [lbs]	Height [ft]	Sex	L. Wrist Circ. [in]	R. Wrist Circ. [in]
1	5	55	130	5 1/3	f	5 3/8	5 5/8
2	5	81	140	5 1/6	f	6 3/4	6 1/2
3	5	74	168	5 4/7	f	6 3/4	7 1/8
4	5	80	139	5	f	8	8
5	5	86	130	4 1/2	f	5	5 1/4
6	4	55	145	5 1/2	f	6	6 1/8
7	4	80	170	5 1/3	m	7	7
8	4	85	153	5	f	5 3/4	5 3/4
9	4	56	138	5 3/7	f	6 3/8	6 1/2
10	4	83	128	5	f	5 3/4	6 1/2
11	4	82	188	5	f	7	7
12	4	69	177	5 3/7	f	7 1/4	7 1/4
13	4	84	140	5 1/6	f	6 3/4	7
14	4	88	138	5	f	6	6
15	4	79	158	4 5/6	f	6 5/8	6 5/8
16	4	76	180	5 1/6	f	8 1/4	8 1/4
17	4	84	150	5	f	5 3/4	5 3/4
18	4	90	134	4 3/4	f	6 1/2	6 1/4
19	4	87	180	5 1/6	f	7 3/8	7 1/2
20	4	80	115	4 5/6	f	5 3/4	6
21	4	79	148	5 1/6	f	6 1/4	7
22	4	78	172	5	f	7 1/2	7 3/4
23	4	72	150	5 1/3	f	5 3/4	6
24	3	56	190	5 1/3	f	6 1/2	6 3/4
25	3	50	155	5 3/7	f	5 1/2	5 3/4
26	3	55	170	5 1/2	f	6 1/4	6 1/4
27	3	58	110	5 1/4	f	5 3/8	5 1/2
28	3	54	145	5 2/3	f	6 1/4	6 1/4
29	3	75	155	5 3/4	m	7	7 1/8
30	3	64	150	5 4/5	m	6 4/5	7
31	3	77	125	5 1/3	f	6	6
32	3	44	120	5 1/4	f	5 1/4	5 3/4
33	3	57	145	5 1/3	f	6 3/8	6 1/4
34	3	56	190	5 1/2	m	7 3/4	8
35	3	44	139	5 3/7	f	6 1/4	6 1/4

36	2	42	156	5	f	6	6
37	2	51	160	5 1/6	f	5 3/8	5 3/4
38	2	50	175	5 3/4	m	6 3/4	6 3/4
39	2	60	160	5	f	7	7
40	2	62	186	5 1/2	m	7 1/2	7 1/2
41	2	63	142	5 1/4	f	6 1/2	6 1/2
42	2	47	218	5 5/6	m	7 3/4	7 3/4
43	2	71	163	5 4/7	f	6 7/8	7
44	2	58	200	5 3/4	m	7	7 1/4
45	2	69	190	5 2/3	m	7 1/4	7
46	2	79	189	5 2/3	f	7 3/4	7 1/2
47	2	74	179	5 1/3	f	7 7/8	7 5/8
48	2	82	188	5	f	7	7
49	2	86	165	5 1/2	f	6 5/8	6 3/4
50	2	67	217	5 1/3	f	7 1/4	7 1/2
51	2	72	180	5 1/4	f	7 1/2	8
52	2	72	185	5 3/7	f	7 1/4	7 1/4
53	2	78	142	5	f	7 1/4	7 1/4
54	2	69	191	5	f	7 1/4	7 3/8
55	2	72	159	5	f	7	7
56	2	72	170	5 1/2	f	7 1/2	8 1/8
57	2	78	201	5 1/6	f	8 1/8	8 1/4
58	2	62	180	5 1/4	f	7 3/8	7 1/2
59	2	80	175	5	f	7	6 5/8
60	2	72	190	5 3/7	f	7 1/8	7 1/8
61	2	70	182	5	f	7 1/2	7 5/8
62	2	69	182	5 1/4	f	7 1/2	7 1/2
63	2	81	150	5	f	6 3/8	6 5/8
64	2	94	156	4 2/3	f	6 3/4	6 7/8
65	2	60	152	5 1/6	f	5 1/4	5 1/4
66	2	60	220	5 1/6	m	7 3/4	7 1/2
67	2	65	180	5 5/6	m	6 3/4	6 7/8
68	1	28	152	5 1/3	f	6 3/4	6 1/2
69	1	29	256	5 3/4	m	8	8 1/4
70	1	30	198	5 3/4	m	7 1/4	7
71	1	23	185	5 5/6	m	6 7/8	6 5/8
72	1	26	124	5 1/3	f	6	6
